# Enabling Genome Editing for Enhanced Agricultural Sustainability

**DOI:** 10.3389/fgeed.2022.898950

**Published:** 2022-05-18

**Authors:** Felicity Keiper, Ana Atanassova

**Affiliations:** ^1^ BASF Australia Ltd., Southbank, VIC, Australia; ^2^ BASF Belgium Coordination Center, Technologiepark-Zwijnaarde, Ghent, Belgium

**Keywords:** agricultural sustainability, plant breeding, crop improvement, genome editing, biotech crops, biotech regulation

## Abstract

Agricultural sustainability encompasses environmental, social, and economic aspects, all of which are continually shifting due changing environmental pressures and societal expectations. A range of strategies are required to address these challenges, and these include the use of innovation and adoption of the best available practices and technologies. Advances in biotechnologies, including genome editing, and their application in plant breeding and research are expected to provide a range of benefits that contribute to all aspects of agricultural sustainability. However, adoption of these technologies needs to be supported by proportionate, coherent, forward-looking, and adaptable policies and regulatory approaches. In this Perspective, we reflect on the regulatory challenges associated with commercialising a transgenic crop, and developments thus far in providing regulatory clarity for genome edited crops. We aim to demonstrate that much remains to be done to shift towards a more proportionate and enabling approach before the potential benefits of genome edited crops can be realised. The implications of precautionary and disproportionate regulation are also discussed.

## Introduction

In 1987, the World Commission on Environment and Development report “Our Common Future” (also known as the Brundtland Report) introduced the concept of sustainable development as “development that ensures that the needs of the present are met without compromising the ability of future generations to meet their own needs”. This concept recognised the interconnected environmental, social, and economic aspects of sustainable development, and also the role of technology in progressing these (United Nations, 1987). If the basic elements of the Brundtland concept are applied to agriculture, sustainable agriculture could be defined as meeting society’s food, feed and fibre needs in the present without compromising the ability of future generations to meet their own needs. It follows that this would consist of production practices that do not compromise environmental integrity and the goods and services provided, as well as socio-economic outcomes, such as profitability along the supply chain, and improved quality of life for society more broadly (Tilman et al., 2002; Pretty, 2007; Allen et al., 2009).

Enhancing agricultural sustainability requires an understanding of the diversity and complexities of agricultural and food systems, and effective and continually adaptable strategies to manage these (Sustainable agriculture, 2018). In the next 20 years, it is expected that agriculture will undergo significant diversification in production systems to meet increasing demand driven by population growth, challenging environmental conditions and climate change, and increasing societal expectations for sustainability. Another important aspect shaping this future is technological innovation, based on advances in genetics, data analysis and automation. Such a diverse agricultural system requires supportive policies that are flexible and rapidly adaptable to change (Bock et al., 2020).

In our view, strategies that support agricultural sustainability include adoption of the best available practices and technologies to support diverse and co-existing production systems, while at the same time reducing, or even enhancing, the environmental footprint (Pretty, 2007; Pretty et al., 2018). Since the 1990s, biotechnological tools have been recognised for their potential to advance plant breeding and crop production levels (e.g., Ruttan, 1999; Ronald, 2011; Federoff and Kershen, 2014; Gartland and Gartland, 2018). It has since been established that the adoption of transgenic (or genetically modified, GM) crops has contributed to improving the sustainability of agricultural practices. For example, increases in crop yields per unit of land, decreased greenhouse gas emissions, decreased reliance on farming inputs, increased insect biodiversity on farms, and increased income for producers have been measured since commercial production began (e.g., Pray et al., 2002; Barrows et al., 2014; Klümper and Qaim, 2014; Brookes and Barfoot, 2015; National Academies of Sciences, Engineering, and Medicine, 2016; Brookes and Barfoot, 2020a; Brookes and Barfoot, 2020b; Kovac et al., 2021; Tokel et al., 2021). Despite the demonstrated benefits, acceptance of transgenic crops remains a social and political flashpoint in many jurisdictions. As a consequence, regulatory approaches vary, and this has contributed to limiting the realisation of the range of potential benefits.

With the uptake of genome editing tools and their application in plant research and breeding (Puchta et al., 2022), there is renewed optimism around opportunities for crop improvement driven by expectations for improved societal acceptance, a more favourable regulatory environment, and reduced cost and time of development. All of these will increase accessibility and adoption of innovative breeding tools in all types of crops (Abdallah et al., 2021; Lassoued et al., 2021). The scientific literature strongly emphasises the features of ease and speed for trait and crop improvement using genome editing, with estimates for halving the development process, e.g., from 8 to 10 years with conventional tools, 8–12 years if transgenic tools are also used, and 4–6 years with the use of genome editing (Chen et al., 2019). Genome editing tools are expected to be widely implemented in plant breeding and to complement existing breeding processes (Jorasch, 2020; Gao, 2021).

Globally, the regulatory landscape for genome edited crops is evolving. Discussions on if/how to appropriately regulate technologies and/or the resulting products are ongoing since early this century. Genome editing presents a regulatory challenge because the types of genetic changes that can be achieved range from sequence insertions that are comparable to transgenics, to mutations indistinguishable from those possible using conventional breeding tools (Jenkins et al., 2021). Reviews of regulatory developments (e.g., Atanassova and Keiper, 2018; Schmidt et al., 2020; Jenkins et al., 2021) show that a growing number of regulatory agencies have provided regulatory clarity for certain uses, categories of technologies and/or types of genetic modifications. Regulators have used either technical adaptations to existing transgenic regulatory frameworks, or enacted new administrative processes, laws, or regulations. We see such changes as the first steps towards more flexible and adaptive regulations, however, much remains to be done to achieve solutions that enable timely uptake of safe new products that have the potential to contribute to agricultural sustainability.

In this article we examine the current complexities of the path to market for a genome edited crop, which to this day remains unclear and uncertain for globally traded commodities. In doing so we aim to demonstrate the important role of a proportionate, coherent, forward-looking, and adaptable regulatory environment in enabling the adoption of innovations in plant breeding and realisation of their potential benefits for agricultural sustainability.

## Regulatory Pathway for Commercialising a Transgenic Crop

**FIGURE 1:**
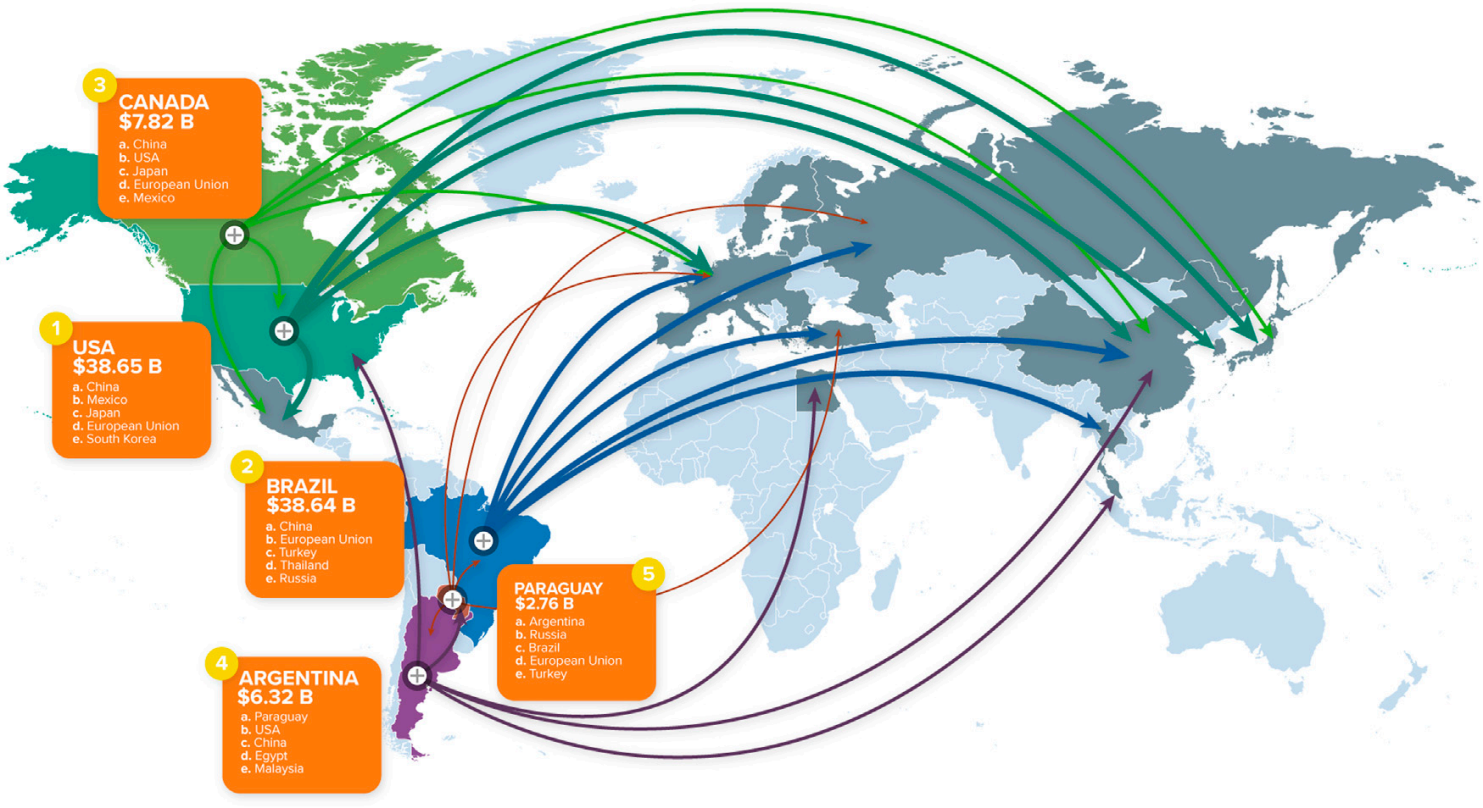
World map showing the top five producers of transgenic crops and their major trading partners, compiled by CropLife International based on 2018 data (source: https://croplife.org/news/global-agriculture-a-trade-map/). More information for each of the top five countries is available at the CropLife International website. Approval was obtained from CropLife International to use the image in Figure 1.

The regulatory approaches used throughout the world for transgenic crops all involve some form of premarket assessment that addresses safety and/or novelty of the product. The approach taken depends on the jurisdiction and the intended use of the product. For example, an environmental risk assessment and regulatory approval are needed to allow planting in a field [small (trial) to large (commercial) scale]. The products of that crop may then be used domestically in the food (human) or feed (animal) supply or in processing (e.g., for food ingredients such as flour), and these uses typically require food/feed safety assessment. The products of that crop may also be exported, and this necessitates the applicable food/feed safety assessments and regulatory approvals in trading partners. To illustrate this global movement, Figure 1 shows the top five transgenic crop producers in the world and their major (not all) trading partners.

The global movement of agricultural biotech commodities relies on regulatory processes in multiple jurisdictions, and these will impact if and when commercial launch of a transgenic crop can occur and under what (if any) conditions. Where the regulatory processes are incomplete or where they do not function well in a jurisdiction, international trade can be disrupted. To mitigate such risks, the major biotech developers have established industry guidelines for commercial launch of transgenic commodity corn (maize), soybean and canola crops,^1^ and best practices in product launch stewardship.^2^ These require the developer to undertake a market and trade assessment to determine key trading partners and the requisite regulatory approvals for commercial launch.

Addressing the applicable regulatory processes for a globally traded agricultural commodity is a significant investment for technology developers. This involves the generation of regulatory studies and navigation of multiple agencies, with many jurisdictions having multiple agencies that manage different aspects of the regulatory assessment, with each agency having its own processes and requirements (Prado et al., 2014; Jenkins et al., 2021). According to data from a survey of CropLife International member companies, the research, development, and commercialisation process for a transgenic crop commercialised in the period 2008–2012 cost USD$136 million with a timeline averaging 13 years. The regulatory component of this amounted to USD$35.1 million (25.8% of the total) and 5.5 years (36.7% of the total) (Phillips McDougall, 2011).

The costs, timelines, and sheer manpower necessary to navigate and obtain a “global registration” of a transgenic crop are major reasons why small-medium enterprises (SMEs) and the public sector have limited their use of transgenic technologies in plant breeding (Strauss and Sax, 2016; Gleim et al., 2020). These are also reasons why the variety of transgenic crops and traits that have been developed, as evident in the scientific literature, greatly exceeds that which have been successfully commercialised (National Academies of Sciences, Engineering, and Medicine, 2016). Transgenic crops in commerce are dominated by three commodity crops (cotton, soybean, maize) and two traits (insect resistance and herbicide tolerance) (National Academies of Sciences, Engineering, and Medicine, 2016; Whelan et al., 2020), as the market opportunity for a developer must be sufficiently large to justify the required investment (Prado et al., 2014). The optimism surrounding the potential of genome editing in plant breeding is juxtaposed against this reality, with realisation of its potential—including a diversity of developers, traits, and crops—requiring relatively risk proportionate regulatory approaches based in science and informed by three decades of experience with transgenic crops.

## Regulatory Pathway for Crops Developed Using Genome Editing

Genome editing can be used to achieve a range of genetic changes in plants, from mutations that are indistinguishable from those arising spontaneously or induced using conventional breeding, to insertions of “foreign”^3^ genetic sequences (Jenkins et al., 2021). The regulatory scenario for commercialising a transgenic crop is also expected to apply to genome edited crops that contain “foreign” genetic sequences. It will also apply more broadly to other applications of genome editing irrespective of the type of genetic change in jurisdictions where regulation of agricultural biotechnology is highly politicised. For example, in New Zealand, revisions to the existing process-based scheme intentionally captured all uses of genome editing in plants within scope (Kershen, 2015), and in the European Union (EU), the scope of definitions in the existing regulatory scheme have been interpreted in this broad way (Hundleby and Harwood, 2018). In both of these jurisdictions, these outcomes are connected to court decisions on questions of scope (Schmidt et al., 2020) and represent the most precautionary regulatory approach.

Regulating indistinctly all types of genome edited crops not only restricts market access, but also presents a major obstacle to investment in their development. In a survey of plant breeding companies in the EU, this was shown to be the case irrespective of the size of the enterprise, with larger developers relocating research and development programs to jurisdictions with more enabling regulatory environments. This refocus to non-EU markets may not be feasible for SMEs with markets predominantly in the EU, who reported discontinuing, modifying or postponing research and development programs that involved the use of genome editing tools (Jorasch, 2020). These issues appear to extend to less politicised jurisdictions where some regulatory clarity has been provided. In a survey of the Canadian plant breeding sector, public sector respondents exhibited less optimism about genome editing (specifically the tool known as CRISPR-Cas9) than private sector respondents. Despite most of the survey respondents expecting a more favourable regulatory environment for genome editing, the public sector remained concerned about regulatory clarity and cost and societal acceptance issues (Gleim et al., 2020).

The regulatory status remains unclear for genome edited crops that are not transgenic, i.e., those that are comparable to conventionally developed crops. For certain technologies and/or the resulting genetic changes in plants, regulatory agencies in Argentina, Australia, Brazil, Canada, Chile, China, Colombia, Ecuador, Guatemala, Honduras, India,^4^ Israel, Japan, Nigeria, Paraguay, Philippines, United States (US), and Uruguay have determined that they are not within the scope of regulatory schemes that apply to transgenic crops, or they may not be within scope depending on a case-by-case assessment of the genetic change (Hundleby and Harwood, 2018; Entine et al., 2021; Lassoued et al., 2021; Mallapaty 2022). Regulatory policy is still evolving in some jurisdictions, e.g., the United Kingdom recently reduced requirements for conducting research trials with genome edited crops (Ledford, 2021), with broader exemptions from transgenic regulation under discussion (UK Parliament, 2022).

One of the listed jurisdictions is Argentina, which is one of the largest producers of transgenic crops (International Service for the Acquisition of Agri-biotech Applications, 2019), and it was one of the first jurisdictions to implement a regulatory framework in 1996 to enable their commercial production (Lema, 2019; Vesprini et al., 2022). In 2015, Argentina became the first country to enact a new regulatory process for determining the status of plants developed using a “new breeding technique” (NBT; includes genome editing) (Normative Resolution No. 173/2015; Lema, 2019). This process involves a case-by-case assessment of whether or not the final product falls within the scope of the established regulatory process for transgenic crops. Four years into its implementation, a notable diversification was observed in the types of developers submitting NBT applications, with greater representation by SME and R&D organisations, as well as in the types of crops and traits developed in comparison to transgenic applications (Whelan et al., 2020). These early observations are consistent with expectations that genome editing will stimulate innovation in crop improvement, and highlight the enabling role of an adaptive regulatory environment in supporting local technology adoption. Subsequently, several countries in Latin America have adopted similar approaches to Argentina, and this will contribute to harmonisation in the region (Turnbull et al., 2021).

Australia is another jurisdiction often listed in the literature as excluding genome editing from transgenic regulation, however this is the case for one technology category, and for one agency, the Office of the Gene Technology Regulator (OGTR). The OGTR regulates environmental releases of transgenic crops, with a long-established regulatory framework and many approvals issued for limited (field trial) and commercial scale releases since 2001.^5^ In 2019, the OGTR expressly excluded site directed nuclease (SDN) applications that do not involve homology directed repair (also known as SDN-1) from regulatory scope, but expressly included site directed nuclease applications that do involve homology directed repair (SDN-2 and SDN-3), as well as oligonucleotide-directed mutagenesis (Gene Technology Amendment (2019 Measures No.1) Regulations 2019; Thygesen, 2019). This means that a crop developed with the use of “SDN-1” can be planted in Australia without assessment or approval by the OGTR. However, before the products of that crop can enter the Australian food supply, developers require resolution of their regulatory status in the food law administered by Food Standards Australia New Zealand (FSANZ) (Kelly, 2019). This in effect limits any potential crop developed with the use of SDN-1 to non-food uses, and it would require management via a closed loop system to prevent it from entering the food supply. Local technology developers and researchers have indicated that this is a consideration impacting technology adoption (Zhang et al., 2021).

To date there are limited examples of commercialised field crops where genome editing was used in their development. One such example is a high oleic soybean that produces a premium cooking oil with less saturated fatty acids compared to commodity soybean oil (Calyxt, 2019). The product is only sold on the US market where it is not regulated as a transgenic crop. It is grown in an identity preserved closed loop system that involves tracking of the entire process from seed to final product.^6^ This enables the technology developer to manage quality of the product, as well as control movement of it and thereby prevent it from entering other markets where the regulatory status is less favourable or unresolved.

## Discussion

This Perspective reflects on the regulatory challenges associated with commercialising a transgenic crop to demonstrate that a shift towards more proportionate policies is necessary to enable the adoption of innovations in plant breeding globally.

To date, restrictive regulatory approaches are evident in jurisdictions where transgenic crops have historically been politicised, with few (or no) approvals granted for commercial planting of transgenic crops. Such jurisdictions have maintained their precautionary process-based approaches that broadly capture products developed with the use of biotechnological tools irrespective of their characteristics and scientific evidence regarding safety. Conversely, relatively proportionate product-focussed approaches have been adopted in certain jurisdictions that have long-term experience in regulating environmental uses of transgenic crops, and who have recognised the potential environmental, social, and economic benefits of technology adoption. An intermediate approach is the selective exclusion or inclusion of certain technological approaches, and while the limited exclusions may be evidence-based, what remains included is on the basis of process and precaution. Of these approaches, the product-based approach has the greatest potential for adapting to continued technological development.

Precautionary process-based approaches to the regulation of transgenic crops have likely contributed to entrenching public distrust of biotechnology (i.e., the process) and a disproportionate perception of risk, rather than allaying concerns. Where such policies are extended to genome editing, a similar outcome can be expected (Herman et al., 2019). The adoption of genome editing in plant breeding provides an opportunity for revising policies in a way that remain consistent with regulatory goals, while also stimulating innovation and enabling broader realisation of potential benefits. We have seen the first steps towards more adaptive policies facilitated by a shift away from process-oriented approaches to a greater focus on the product. This shift has been informed by almost three decades of experience with the assessment and use of transgenic crops and observation of their benefits. However, we remain far from an evidence-based model that links innovation to sustainability in agriculture.

## Data Availability

The original contributions presented in the study are included in the article/Supplementary Material, further inquiries can be directed to the corresponding author.

## Footnotes

1 CropLife International: https://croplife.org/plant-biotechnology/stewardship-2/product-launch-stewardship/.

2 Excellence Through Stewardship: https://www.excellencethroughstewardship.org/.

3 The authors are aligned with the view that “foreign” refers to sequences originating from a non cross-compatible species.

4 For India: ttp://db.zs-intern.de/uploads/1649254604-office%20memorandum%20indien%20genome%20editing.pdf.

5
https://www.ogtr.gov.au/what-weve-approved/dealings-involving-intentional-release.

6
https://calyxt.com/wp-content/uploads/2020/02/Calyxt_GrowerProgram.pdf.
